# Capillary-Electrophoresis-Based Species Barcoding of Big Cats: CR-mtDNA-Length Polymorphism

**DOI:** 10.3390/life14040497

**Published:** 2024-04-11

**Authors:** Lenka Vankova, Daniel Vanek

**Affiliations:** 1 Institute for Environmental Sciences, Charles University, 128 00 Prague, Czech Republic; lenka.vankova@fdnas.cz; 2 Forensic DNA Service, Budinova 2, 180 81 Prague, Czech Republic; 3 Department of Forensic Medicine, Second Faculty of Medicine, Charles University, 128 00 Prague, Czech Republic; 4 Bulovka University Hospital, 180 81 Prague, Czech Republic

**Keywords:** CITES, traditional Chinese medicine, DNA identification, species identification, DNA barcoding, NUMTs

## Abstract

This study aimed to provide an overview of the methodological approach used for the species determination of big cats. The molecular system described herein employs mitochondrial DNA control region (CR-mtDNA)-length polymorphism in combination with highly sensitive and precise capillary electrophoresis. We demonstrated that the described CR-mtDNA barcoding system can be utilized for species determination where the presence of biological material from big cats is expected or used as a confirmatory test alongside Sanger or massive parallel sequencing (MPS). We have also addressed the fact that species barcoding, when based on the analysis of mtDNA targets, can be biased by nuclear inserts of the mitochondrial genome (NUMTs). The CR-mtDNA barcoding system is suitable even for problematic and challenging samples, such as hair. CR-mtDNA-length polymorphisms can also distinguish hybrids from pure breeds.

## 1. Introduction

The illegal trade of endangered species of wild fauna and flora threatens many species. Numerous traditional Chinese medicine (TCM) treatments involve the use of wildlife products, including some that utilize ingredients derived from endangered flora and fauna [1], regardless of whether those organisms are protected by the Convention on the International Trade in Endangered Species (CITES) [2]. The investigation of wildlife crimes requires precise scientific species determination techniques. DNA barcoding [3] seems to be an optimal tool for the detection of source organisms in TCM products. DNA barcoding sensu stricto means that species identification is performed using one standardized DNA fragment. The definition of DNA barcoding sensu lato is not very restrictive and corresponds with any taxonomic-level identification using any DNA fragment [4]. DNA barcoding of the biological material used for the preparation of TCMs has been described for numerous animal [5,6,7,8] and plant [9,10,11,12] species. Non-human DNA typing for forensic purposes is also utilized for the identification of domesticated animals, such as cats [13] and dogs [14], or CITES organisms, such as elephants [15], pangolins [16], rhinos [17], and tigers [18].

Standard Sanger sequencing is not suitable for samples containing biological material from multiple species; thus, massively parallel sequencing must be used instead [19]. Mitochondrial DNA control region length polymorphisms are an alternative to sequencing and can serve as an additional or confirmatory test. PCR amplification using universal primers with subsequent restriction cleavage (PCR-RFLP) can be used to separate species using characteristic patterns [20], but the use of this method is limited by the existence of restriction sites and rather difficult interpretation. Species barcoding can also be achieved using the RAPD approach [21], AFLPs [22], species-specific PCR [23], the SNaPshot assay [24], MRMA analysis [25], or qPCR assays [26]. Another type of barcoding methodology utilizes interspecies insertions/deletions, where the target region is a hypervariable mtDNA D-loop [27], the highly variable regions 12S rRNA 16S rRNA [28], and the control region (CR-mtDNA) length polymorphisms [29].

**Figure 1:**
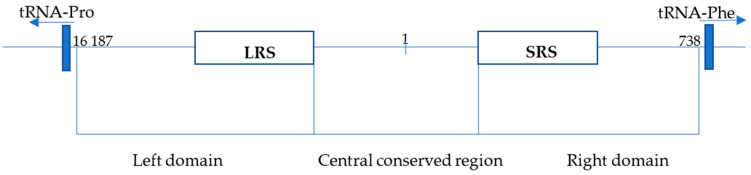
Structure of the control region (CR) of the *Panthera tigris* isolate PTI. The CR of *P. tigris* isolate PTI is located between tRNA-Pro and tRNA-Phe and is 1345 bp long (position 16 187-738). CR is divided into 3 parts: the left domain, with a long repetitive sequence of 80 bp (LRS; position 16 385-16 545); the central conserved domain (position 16 546-16 793, 1-273); and the right domain, with a short repeat sequence of 8 bp (SRS; position 273-377) (based on Bagatharia [33] and Lopez [34]).

This study aims to provide an overview of the methodological approach used by our laboratory for species determination of big cats in our wildlife crime casework [30]. All species of big cats, including tigers, lions, leopards, cheetahs, and jaguars, are protected under the CITES convention but are poached in large numbers to serve as an article of illegal trade comprising bones, teeth, hide, and TCM products that are derived from these species [31,32]. The advantage of the CR-mtDNA barcoding system is its ease of use even in laboratories not performing sequencing techniques. The described assay can be utilized for species determination where the presence of biological material from big cats is expected or used as a confirmatory test alongside Sanger sequencing, MPS, or non-DNA barcoding methods. The molecular system described herein employs mitochondrial-DNA-control-region-length polymorphism (CR-mtDNA) [29] in combination with highly sensitive and precise capillary electrophoresis (see Figure 1).

Species barcoding, when based on the analysis of mtDNA targets, can be biased by NUMTs because the NUMT and the true mtDNA target sequence diverge for the focal taxon [35,36]. To address this issue, we employed exonuclease V to remove the NUMTs’ signal.

## 2. Materials and Methods

The reference material used for this study was provided by the Czech Environmental Inspectorate and zoological gardens. The biological material came either from animals that died from natural causes in zoological gardens, or their excrements. The sampling did not involve the infliction of trauma to living animals. The research thus did not fall under the Directive 2010/63/EU of the European Parliament and of the Council of 22 September 2010 on the protection of animals used for scientific purposes.

Human DNA samples were sampled and used in accordance with the Regulation (EU) 2016/679 of the European Parliament and of the Council of 27 April 2016 on the protection of natural persons with regard to the processing of personal data and on the free movement of such data, following Directive 95/46/EC (General Data Protection Regulation).

DNA extraction from the reference material (see Table 1) was performed using a Quick-DNA Microprep Plus Kit (ZymoResearch, Irvine, CA, USA) with final elution to 20 µL H_2_O. The quantification of extracted DNA was achieved using UV–VIS or qPCR [18], where the nuclear DNA concentrations were measured by targeting the STR locus Pati01 [29].

### 2.1. PCR Composition and PCR Conditions for Fragment Analysis

The primers used for the amplification of mitochondrial-DNA-control-region-length polymorphisms have been described by Pun et al. [29]. Primer sequences: L15995 5′CTCCACTATCAGCACCCAAAG 3′; H16498 5′CCTGAAGTAAGAACCAGATG 3′.

The PCR mixture contained 1.25 μL of GoldStar 10× buffer (Promega, Madison, WI, USA), 10 μM L15995 + H16498 and 0.5 + 0.5 μL, and the primer L15995 was fluorescently labeled with 5-FAM, 0.25 μL (5 U/μL) of AmpliTag Gold DNA polymerase (Applied Biosystems, San Francisco, CA, USA), 10 pg of DNA, and H_2_O to a final volume of 12.5 μL. The PCR program was as follows: 95 °C for 10 min, 32× (95 °C for 15 s, 55 °C for 30 s, 72 °C for 1 min), and 72 °C for 30 min.

PCR was performed using a MasterCycler Nexus gradient thermocycler (Eppendorf, Germany). The resulting amplicons were visualized using capillary electrophoresis (SeqStudio 3200 Genetic Analyzer; Applied Biosystems, USA) under the following parameters: G5 matrix, 12 µL formamide, 0.4 µL LIZ 1200 (Applied Biosystems, USA), 1 µL of PCR product. Raw data processing was performed using GeneMapper5 (Applied Biosystems, USA).

### 2.2. PCR Composition and PCR Conditions for Agarose Gel Electrophoresis and Sanger Sequencing

The PCR mixture contained 10× Gold buffer (Applied Biosystems, USA), 2 μL of 25 mM MgCl_2_, 0.5 μL of 10 mM dNTPs, 0.5 + 0.5 μL of 10 μM L15995 + H16498, 0.2 μL of 5 U/μL AmpliTag Gold DNA polymerase (Applied Biosystems, USA), 0.1–1 ng of DNA, and H_2_O to a final volume of 25 μL. The PCR program was as follows: 95 °C for 10 min, 40× (95 °C for 15 s, 55 °C for 30 s, 72 °C for 1 min), and 72 °C for 30 min.

PCR was performed using a MasterCycler Nexus gradient thermocycler (Eppendorf, Germany). The resulting amplicons were visualized using agarose gel electrophoresis. DNA clean-up of the amplified fragments excised from the gel was performed using a Zymoclean Gel DNA recovery Kit (ZymoResearch, USA). Sanger sequencing was performed using a SeqStudio 3200 Genetic Analyzer (Applied Biosystems, USA). Raw data processing was performed using Sequencing Analysis Software v6.0 (Applied Biosystems, USA).

### 2.3. The Effect of Exonuclease V Treatment

Exonuclease V (RecBCD) (New England Biolabs, Ipswich, MA, USA) was used as suggested by the manufacturer.

To ensure nDNA removal, the molecular system Pleo Qplex was used. This system is based on quantitative RT-PCR with TaqMan probes and primers highly specific for *Panthera leo* (Forensic DNA Service, Prague, Czech Republic). The assay was performed in a single tube/well. Nuclear DNA concentration was measured using the STR locus Pati01 [29], and the concentration of mitochondrial DNA was measured using primers and probes targeted to the Cytochrome B gene of *P. leo*. Quantitative PCR was performed using QuantStudio 5 (Applied Biosystems, USA).

The assay validation followed the ANSI/ASB standard for the internal validation of forensic DNA analysis methods, as described by Webster et al. [37] (see Results section for validation). 

## 3. Results

**Figure 2:**
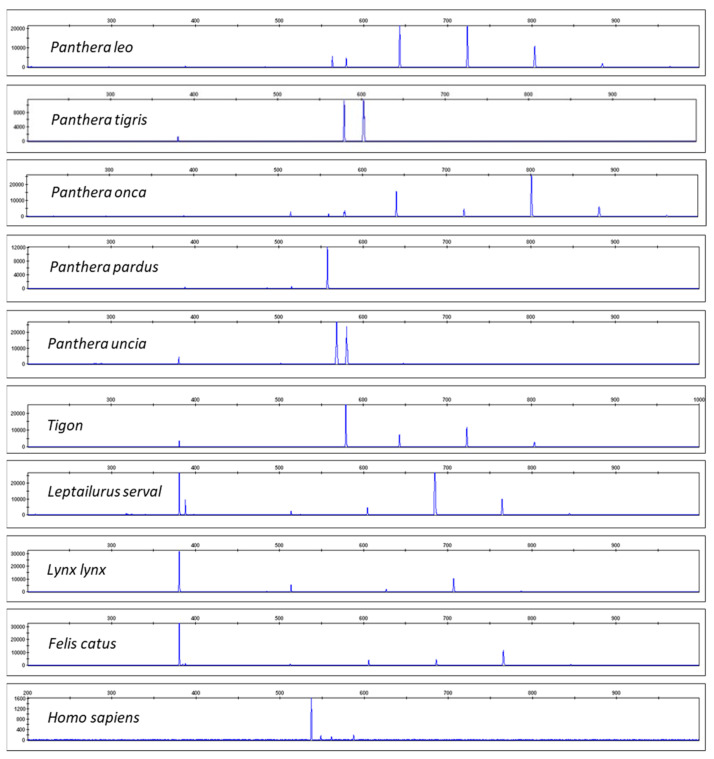
CR-mtDNA electropherograms for *P. leo*, *P. tigris*, *Panthera onca*, *Panthera pardus*, *Panthera uncia*, *Tigon* (hybrid of *P. tigris* and *P. leo*), *Leptailurus serval*, *Lynx lynx*, *Felis catus*, and *Homo sapiens*. Note that all of the species (including the hybrid tiger–lion species) have distinct barcodes.

Figure 2 shows the resulting electropherograms for *Panthera leo*, *Panthera tigris*, *Panthera onca*, *Panthera pardus*, *Panthera uncia*, *Tigon* (a combination of *P. tigris* and *P. leo*), *Leptailurus serval*, *Lynx lynx*, *Felis catus*, and *Homo sapiens*. Differences in the resulting CR-mtDNA barcoding profiles should be noted. We tested multiple individuals per species, including the subspecies *P. tigris* sumatrae, *P. tigris* jacksoni, *P. tigris* altaica, and *P. tigris* ussuri. For details of the samples’ origin see Materials and Methods. The species assignment was also confirmed by Sanger sequencing of COI and cytB mtDNA genes with a subsequent comparison with databases BOLDsystems [38] or GenBank [39]. All resulting DNA CR-mtDNA-length profiles were specific for a given species (see the Supplementary Materials).

**Figure 3:**
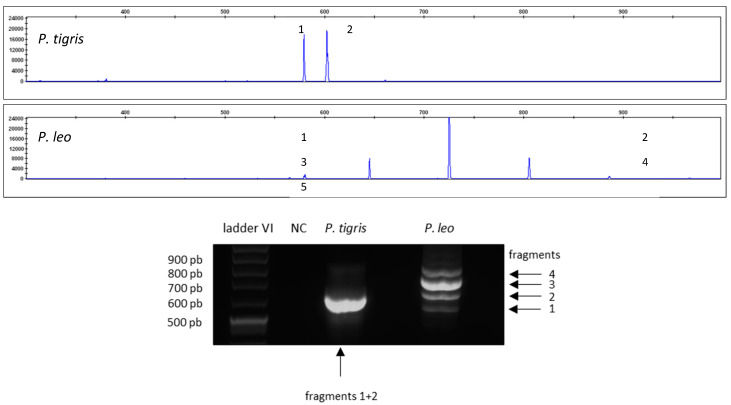
Resulting electropherograms for CR-mtDNA-length polymorphism amplifications of *P. tigris* and *P. leo* analyzed via capillary electrophoresis (**upper panel**) and agarose gel electrophoresis (**lower panel**). Peak 5 is not visible on the agarose gel due to lower sensitivity.

Figure 3 shows the different barcode patterns for *P. tigris* and *P. leo* analyzed via capillary electrophoresis (upper panel) and agarose gel electrophoresis (lower panel).

**Figure 4:**
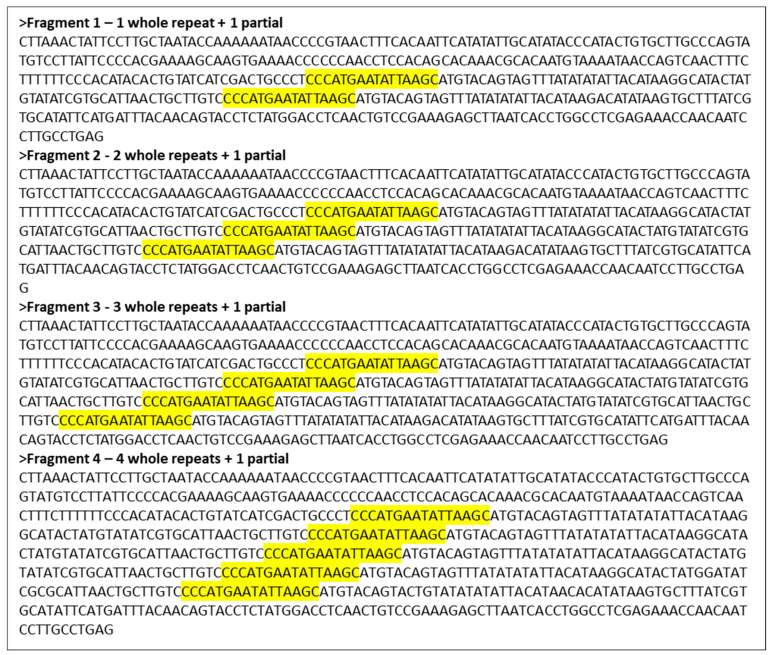
The sequence of *P. leo* CR-mtDNA-length polymorphism amplicons 1–4 (see lower panel of Figure 3), purified from agarose gel. The start of the 80 bp repetitive sequence is marked in yellow.

Figure 4 shows the sequence of *P. leo* CR-mtDNA amplicons 1–4 (see lower panel of Figure 3), which were purified from agarose gels. The start of the 80 bp repetitive sequence (LRS) is marked in yellow.

**Figure 5:**
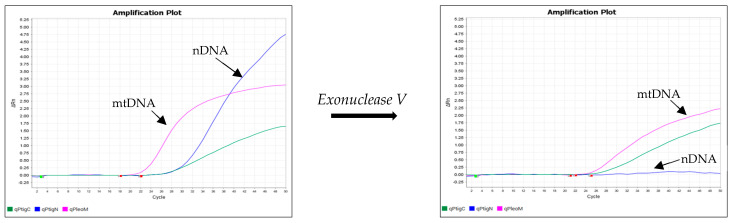
Effect of exonuclease V treatment on DNA isolates, as demonstrated by the multiplex qPCR assay Pleo Qplex, which targets nuclear DNA (blue), mitochondrial DNA (pink), and internal positive control DNA (green).

**Figure 6:**
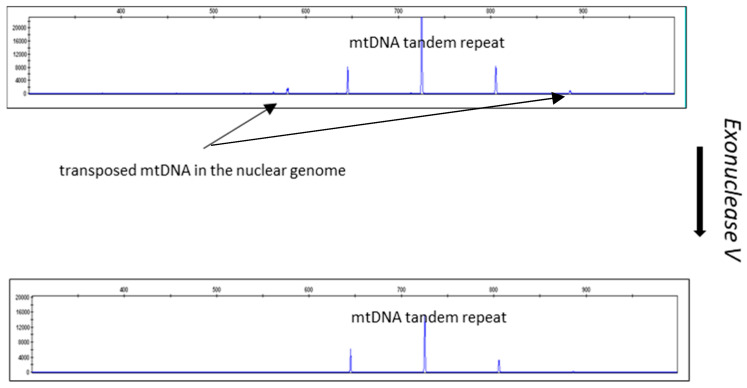
Effect of exonuclease V treatment on DNA isolates visualized by capillary electrophoresis of CR-mtDNA-length polymorphism *P. leo* amplicons. Please note the disappearance of the NUMTs’ peaks (1 and 5). The sample used for the exonuclease V treatment differs from the sample used for the CR-mtDNA electropherograms shown in Figure 2. The mtDNA peaks remaining after ExoV treatment are species-specific.

Figure 5 and Figure 6 show the results of an experiment in which the DNA isolates were treated with exonuclease V. The effect of exonuclease V was demonstrated by the multiplex qPCR assay Pleo Qplex (Figure 5), which targets nuclear DNA, mitochondrial DNA, and IPC (internal positive control), or by capillary electrophoresis (Figure 6).

### Results of the Validation

Specificity: We applied the assay to DNA extracts of closely related species and species that may co-occur (species regularly processed in a lab) or be substituted for the target species (e.g., in TCMs) (*Sus scrofa*, *Bos taurus*, and *C. lupus* on top of the species listed in Table 1). The CR-mtDNA-length polymorphism assay was found to be species-specific with the following exceptions: testing of *P. pardus* subspecies produces slightly different patterns and *P. onca* can be misinterpreted as *P. leo* if only peak positions (without peak heights) are considered. 

Sensitivity: Analytical sensitivity was tested on serial dilutions of DNA from seven target animals frequently occurring in our casework (*P. tigris*, *P. leo*, *P. onca*, *P. pardus*, *L. lynx*, *H. sapiens*, and *F. catus*), with the DNA input ranging from 2 ng to 5 pg. The validation showed that the method’s sensitivity is applicable to DNA inputs of more than 5 pg nDNA (qPCR quantitation results).

Robustness: We tested the robustness of the assay under temperatures shifted against the original protocol (annealing temperature: 55 °C). Changes in the annealing temperature greater than +2 °C will cause the assay to fail to generate the characteristic CE profile.

Repeatability: DNA extracts from different individuals from the target species (*P. tigris* (10), *P. leo* (10), *P. pardus* (5), *P. uncia* (3), *L. serval* (4), *Tigon* (2), and *L. lynx* (4)) were used to test the repeatability (see the Supplementary Material). The results were identical for all of the samples tested, except for the *P. pardus* subspecies which produced slightly different EPGs. 

Reproducibility: We tested the within-lab reproducibility with two different analysts independently running replicates of the assay. The resulting EPGs were identical for all of the samples tested.

Negative and positive controls were run in all of the above experiments and produced results as expected.

## 4. Discussion

Species identification is a crucial aspect of solving criminal cases involving non-human biological materials. Identifying the species can help to narrow down the range of suspects and increase the chances of identifying the true culprit. Currently, there are many techniques based on mtDNA that allow for species identification. However, this relatively streamlined approach, relying on mtDNA typing, becomes challenging when a lab faces a mixture of DNA from different species that can be degraded [40]. When samples contain a mixture of DNA from multiple species, interpreting sequencing analysis results is difficult and requires either a special interpretation framework [41] or a special software deconvolution tool [42]. DNA metabarcoding using next-generation sequencing (NGS) technologies can overcome the DNA mixture problem [43,44,45,46], but it is not suitable for small-scale analyses routinely performed by local forensic laboratories, primarily because it is costly and time-consuming. DNA barcoding of mixed samples using NGS can overestimate the number of species when nuclear mitochondrial pseudogenes are coamplified and sequenced [35,47,48].

PCR analysis enables species barcoding from minute samples, but the number of identifiable taxa is limited when compared with sequencing techniques [40]. The scientific literature describes a relatively small number of species-specific nuclear markers. The banding pattern of the Alu I-digested prepro-gonadotropin-releasing hormone gene is species-specific in Atlantic salmon and brown trout and can be applied for the identification of their hybrids [49]. Trematomid fishes can also be identified using a species-specific nuclear marker [50]. Studies into transposable elements provide some leads for phylogenetic and population genetic investigations [51], but their use is limited. High-resolution melting analysis of the universal ITS2 region of genomic rDNA has been employed using the novel authentication approach for coffee beans and brewed beverages [52]. Unfortunately, none of these nuclear markers would mitigate the limits of the current barcoding system based on mtDNA barcoding. Nuclear markers should be universally applicable across the majority of species and carry sufficient information to discriminate between closely related species [53]. An analysis of metazoan-level universal single-copy orthologs (metazoan USCOs) shows that this approach might also be applicable outside the metazoans [54].

Nuclear inserts of mitochondrial DNA (NUMTs) have been found in major clades of eukaryotic organisms, but the number of NUMTs can significantly vary between different species [55,56,57,58,59], the distribution of NUMTs is lineage-specific, and the presence of NUMTs cannot be known a priori.

Barcoding analysis of mtDNA targets can be biased by NUMTs [35,36]; however, NUMTs can also be used as population genetic markers, e.g., in gorillas [60]. We have demonstrated that exonuclease V treatment can be used to remove nuclear DNA prior to PCR. Other possibilities are to use selective enrichment [61], design the mtDNA barcoding primers in a way that the amplification of NUMTs is greatly reduced [62], use long-range amplification, or use pre-PCR dilution. NUMT sequences are also available in public databases and are wrongly identified as authentic mtDNA [63]. This cannot only cause problems in phylogenetic studies [64] but also in forensic applications [59,65].

## 5. Conclusions

When a confiscated TCM artifact indicates the presence of a CITES organism, this is considered legal proof of its presence. Unlabeled and suspicious TCM artifacts must be sent to the forensic laboratory for species determination. The described CR-mtDNA polymorphic region can be utilized for species determination when the presence of biological material from big cats is expected or used as a confirmatory test alongside Sanger sequencing, MPS, or non-DNA barcoding methods [66,67,68,69]. The advantage of the CR-mtDNA barcoding system is its ease of use even in laboratories not performing sequencing techniques. The polymorphic CR-mtDNA system has relatively short amplicons, and its main advantage is its high sensitivity and thus suitability even for problematic samples, such as hair or tanned hide. The above-described assay has been successfully applied as a confirmatory test for a numerous number of casework samples. The system is sufficiently informative for big cats and does not show intraspecies variability. However, it is necessary to point out that the testing of *P. pardus* subspecies produces slightly different patterns, and *P. onca* can be misinterpreted as *P. leo* if only peak positions (without peak heights) are considered. The greatest disadvantage of mtDNA species barcoding assays is that they do not enable the differentiation of hybrids (for instance, tigon = male tiger and female lion) but rather the female line only [70]. CR-mtDNA-length polymorphism typing, as described herein, can differentiate not only different species of big cats but also hybrids (see Figure 2). Species identification is an important component of forensic science, and the development of new techniques or the upgrading of existing ones will continue to improve the accuracy and efficiency of species identification.

## Figures and Tables

**Table 1:** Species analyzed in this study.

Species	No. of Samples Tested	Sample Type (Number)
*Panthera leo*	30	hair (15), blood (15)
*Panthera tigris*	30	hair (15), tissue (5), blood (5), excrement (5)
*Panthera onca*	2	hair (2)
*Panthera pardus*	10	hair (7), blood (3)
*Panthera uncia*	3	hair (3)
*Tigon* (*P. tigris x P. leo*)	2	hair (2)
*Leptailurus serval*	4	hair (4)
*Lynx lynx*	4	hair (2), excrement (2)
*Felis catus*	2	buccal swab (2)
*Homo sapiens*	5	buccal swab (5)

## Data Availability

Data are contained within the Supplementary Materials.

## Supplementary Materials

The following supporting information can be downloaded at https://www.mdpi.com/article/10.3390/life14040497/s1, *Panthera leo*–samples 1–12; *Panthera tigris*—samples 1–12; *Panthera pardus*—samples 1–4; *Panthera onca*—samples 1–2; *Panthera uncia*—samples 1–2; *Tigon*—samples 1–2; *Lyns lynx*—samples 1–4; *Leptailurus serval*—samples 1–4; *Homo sapiens*—samples 1–2.
