# Supplementary material for: Capillary-Electrophoresis-Based Species Barcoding of Big Cats: CR-mtDNA-Length Polymorphism
Source: Life (Basel). 2024 Apr 11;14(4):497. doi: 10.3390/life14040497 (PMC11051001; doi:10.3390/life14040497)

Panthera leo – samples 1-4

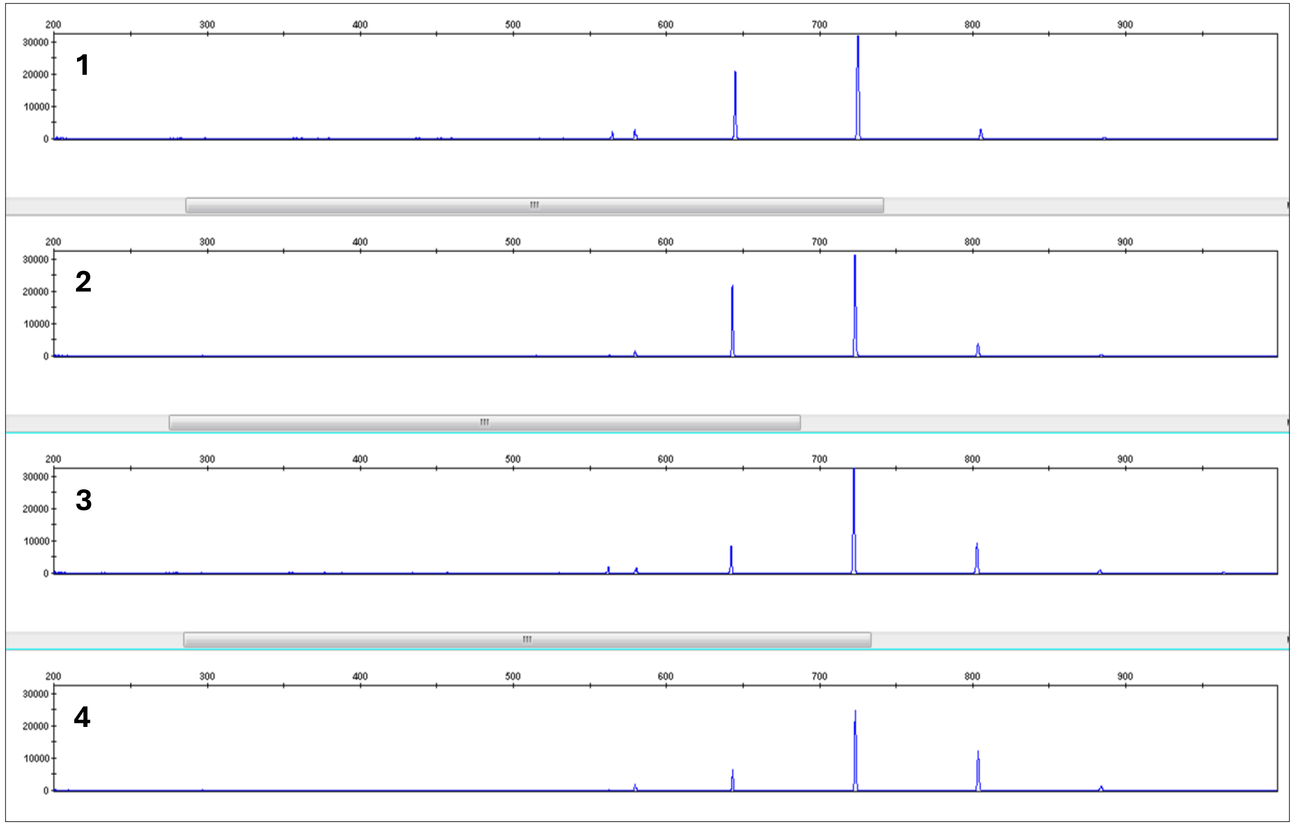

Panthera leo – samples 5-8

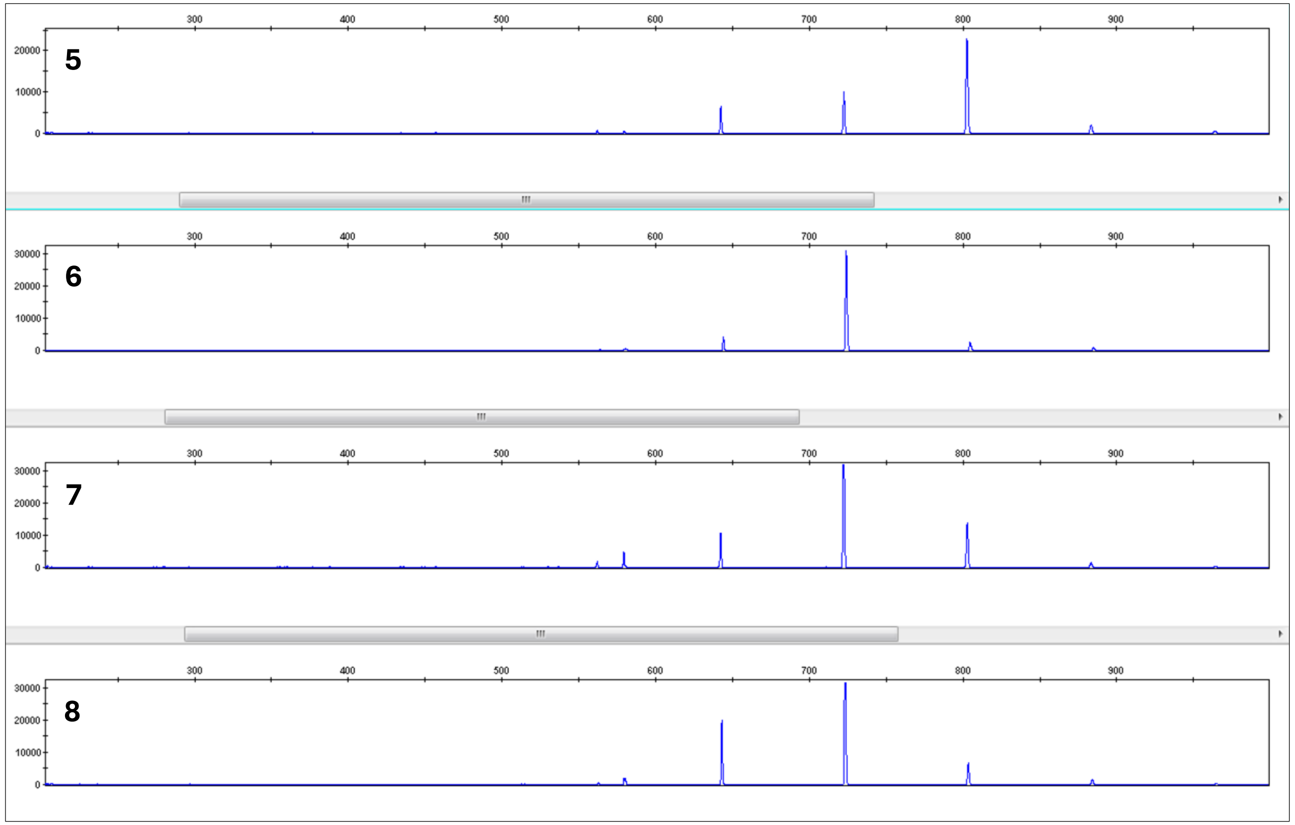

Panthera leo – samples 9-12

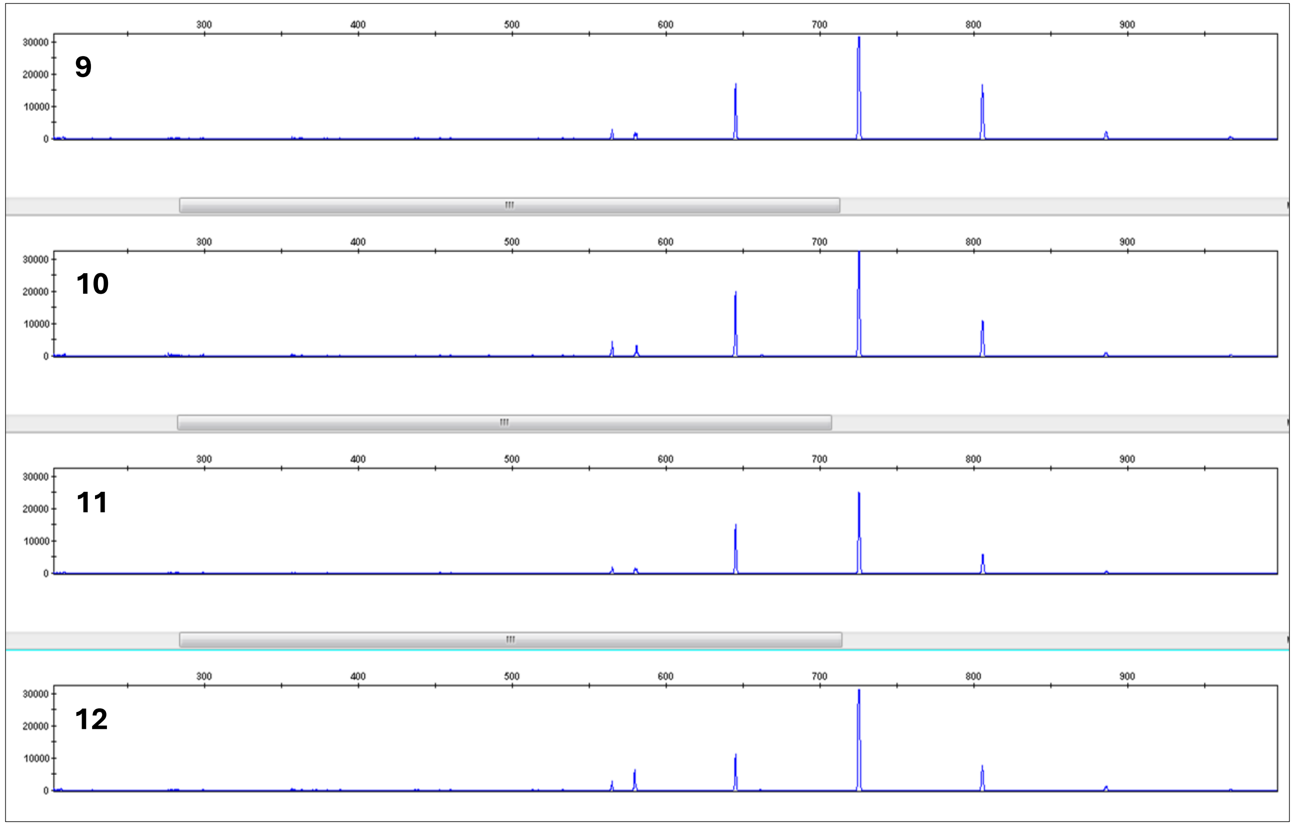

Panthera tigris – samples 1-4

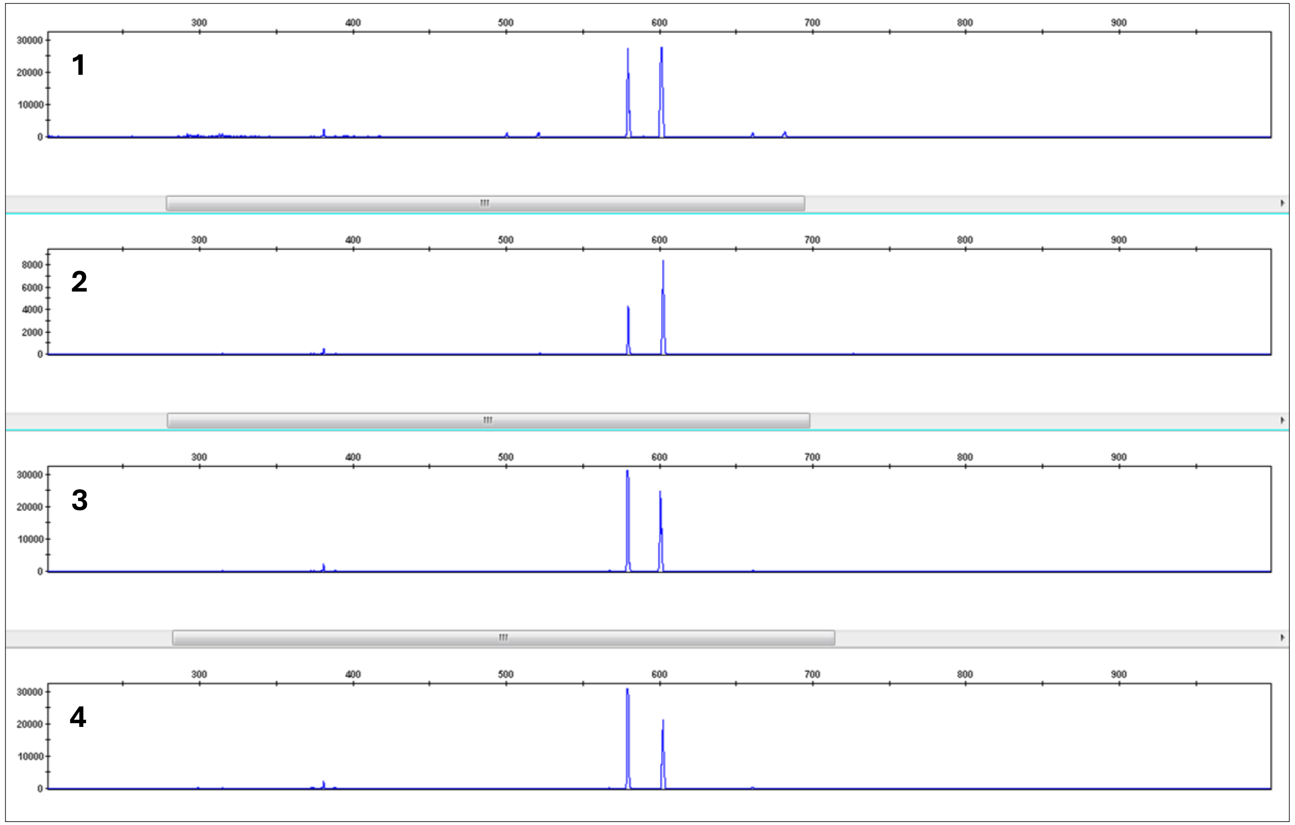

Panthera tigris – samples 5-8

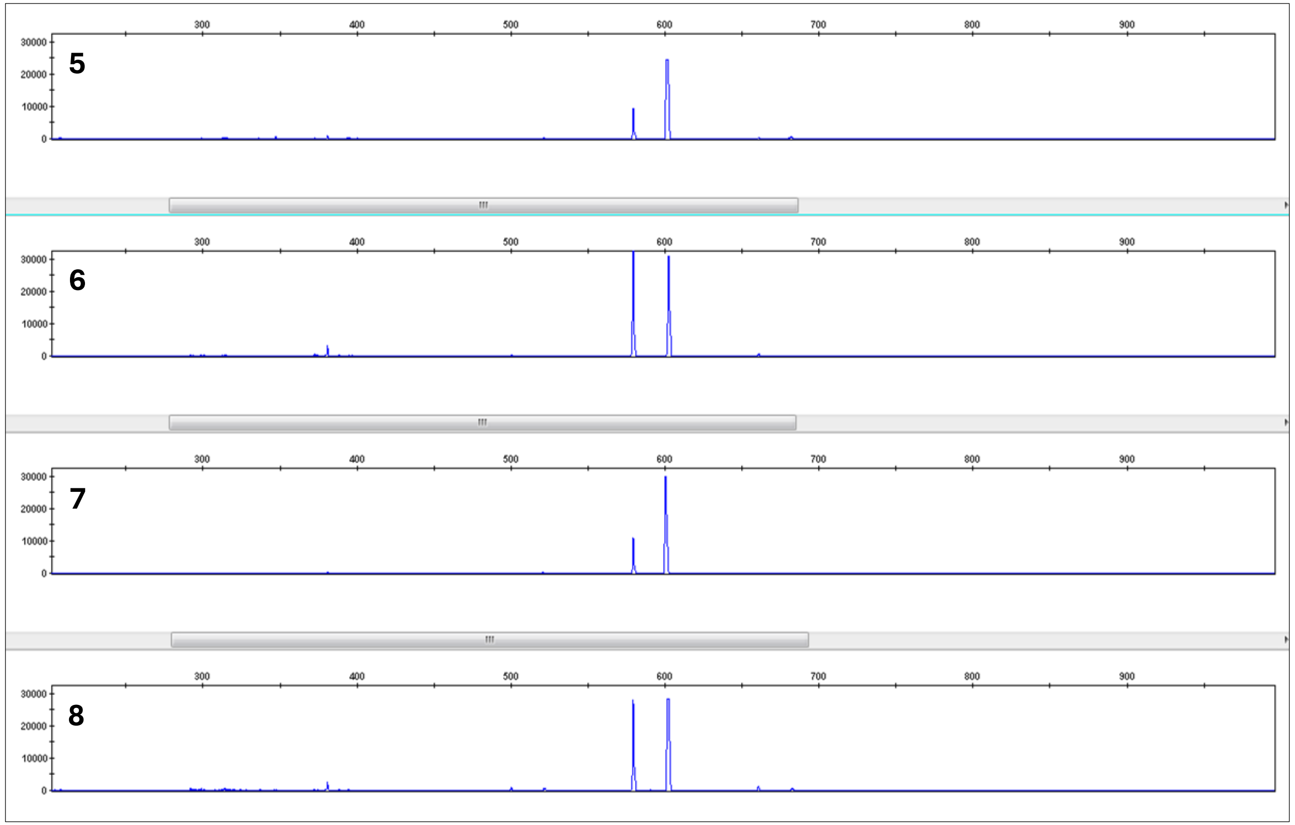

Panthera tigris – samples 9-12

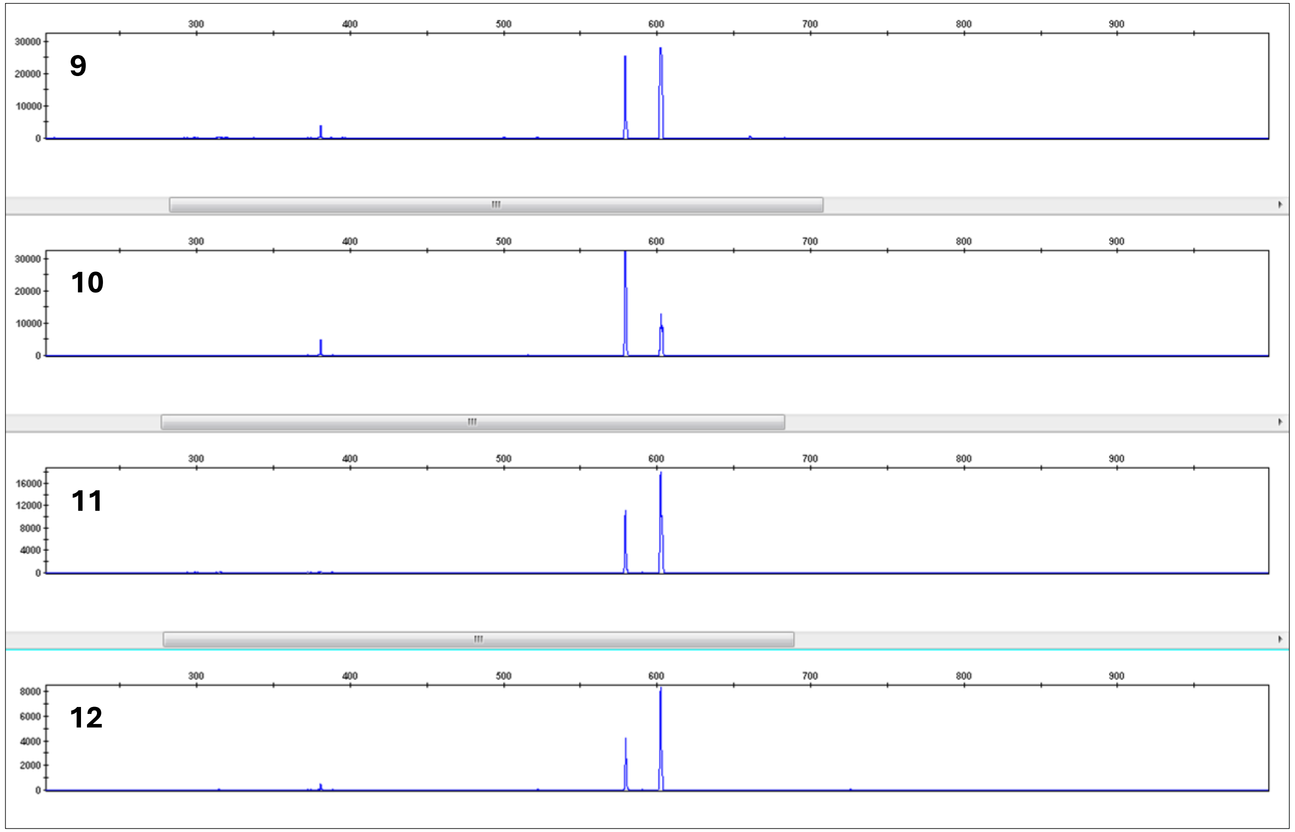

Panthera pardus – samples 1-4

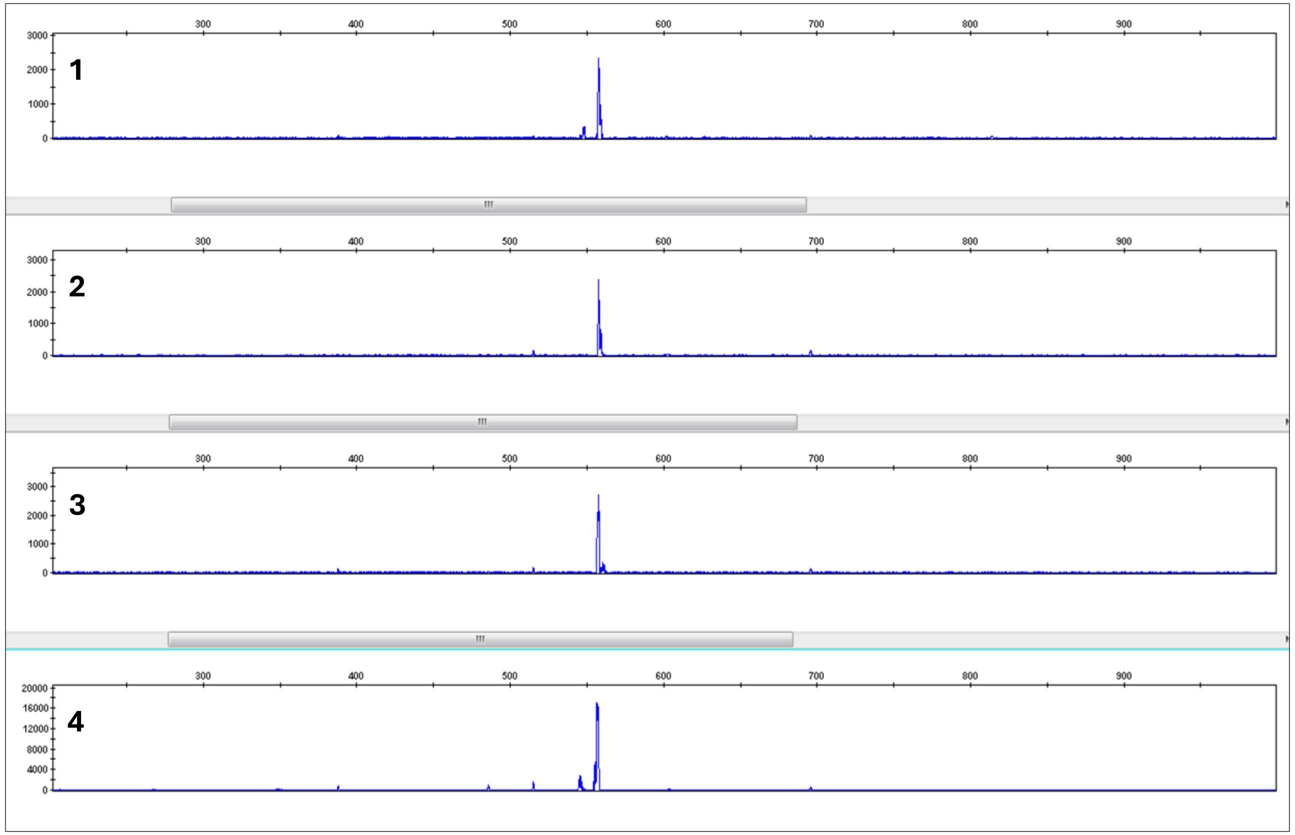

Panthera onca – samples 1-2

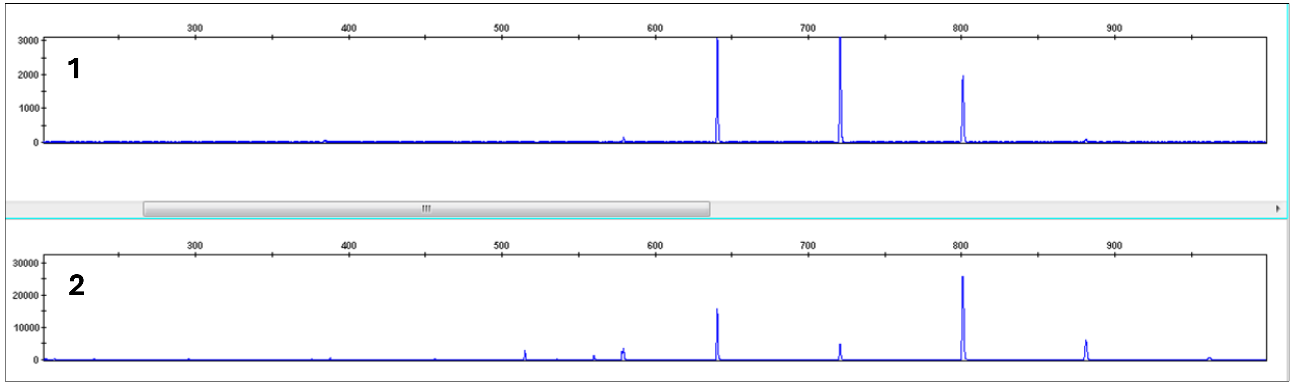

Panthera uncia – samples 1-2

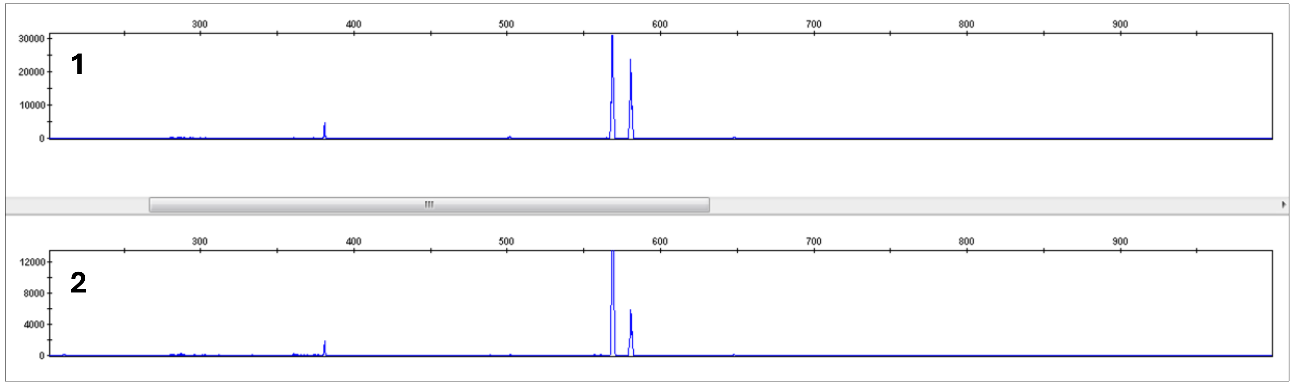

Tigon – samples 1-2

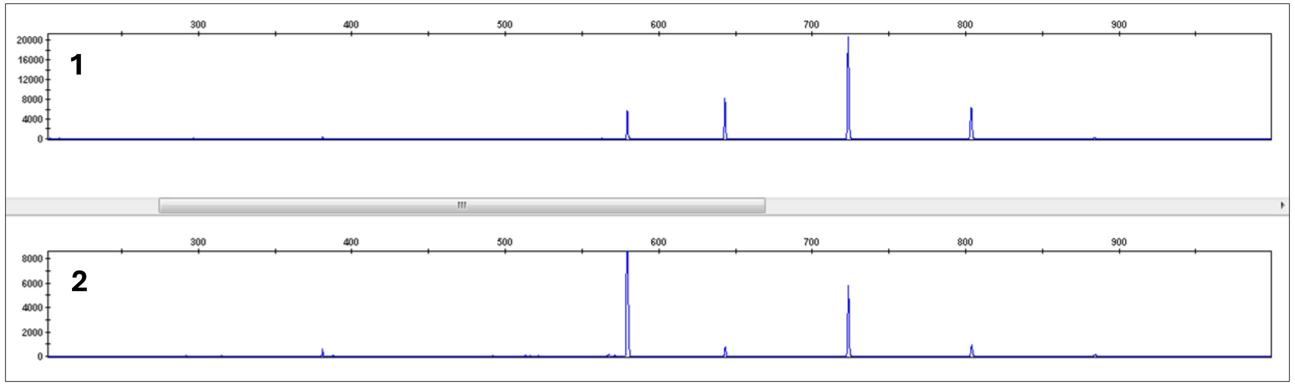

Lynx lynx – samples 1-4

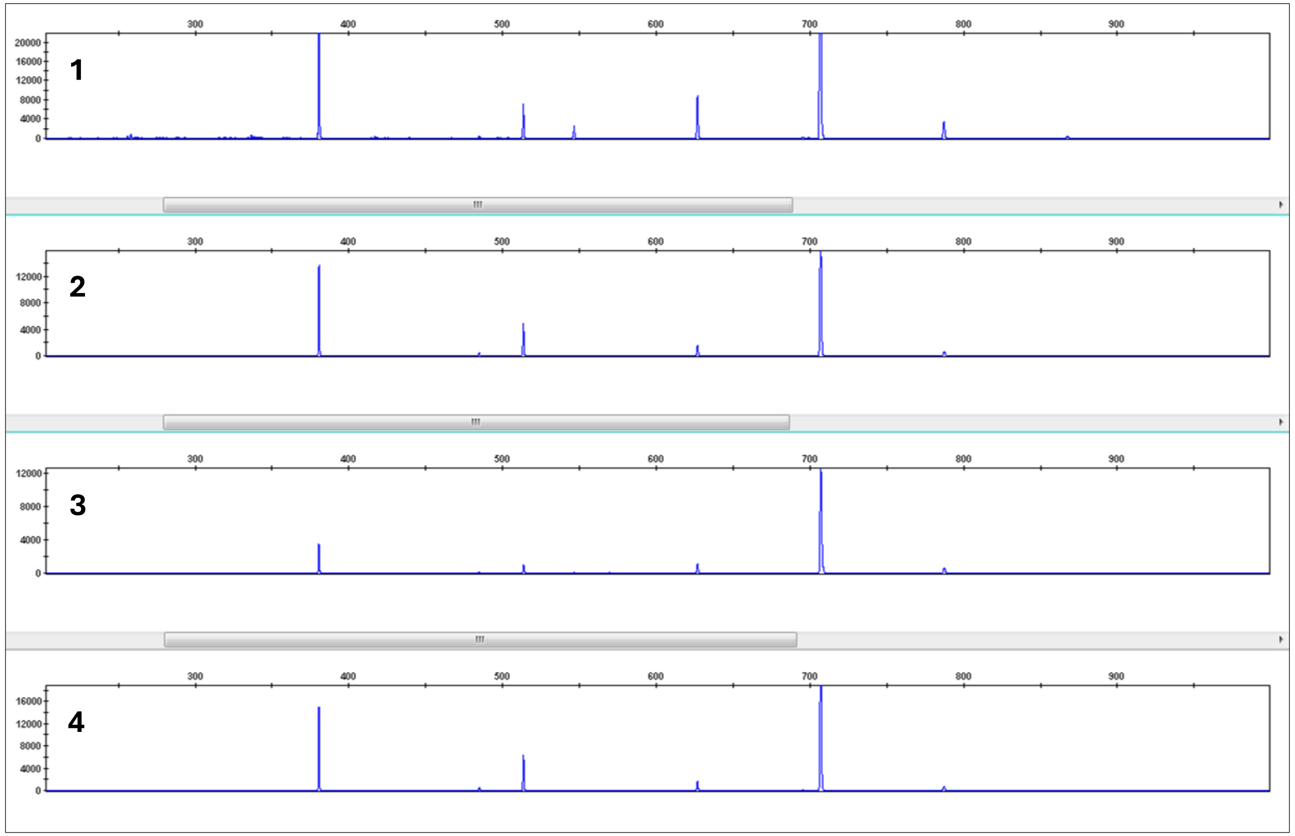

Leptailurus serval – samples 1-4

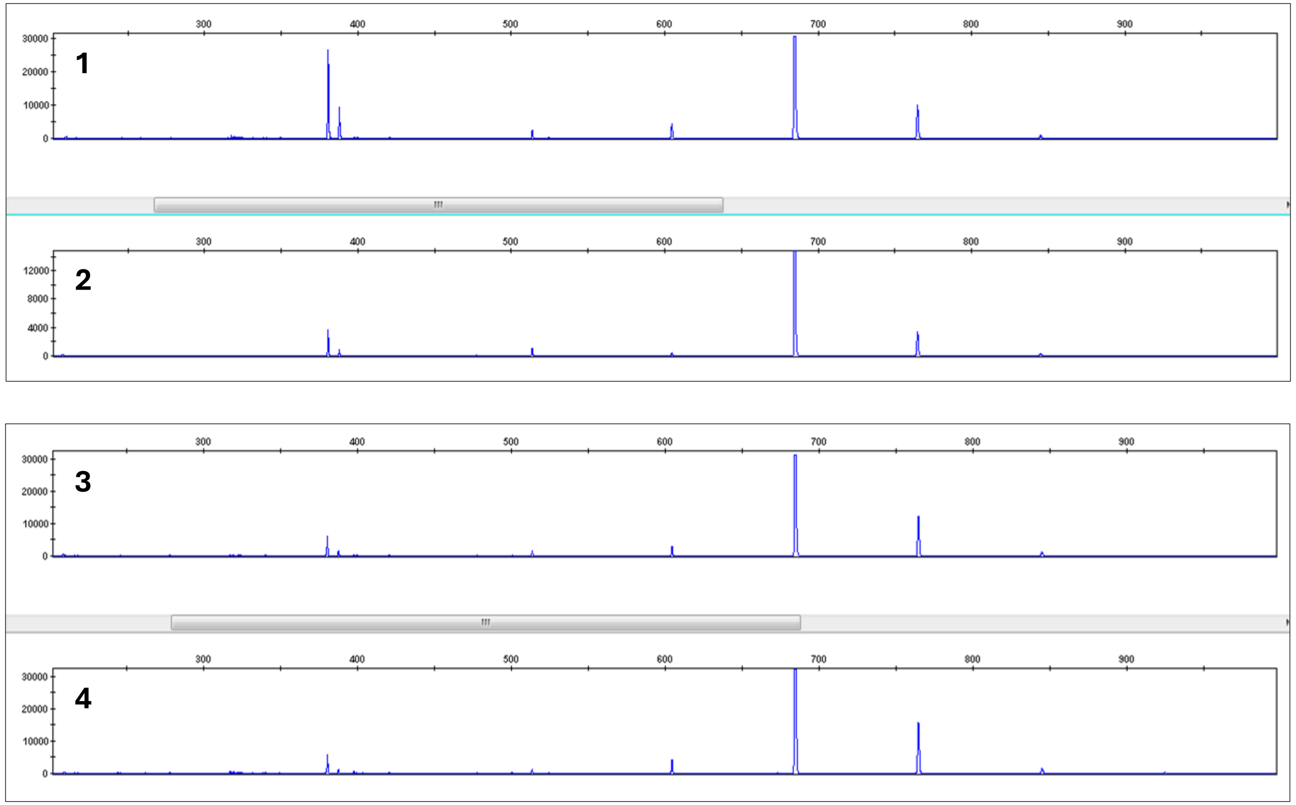

Homo sapiens – samples 1-2

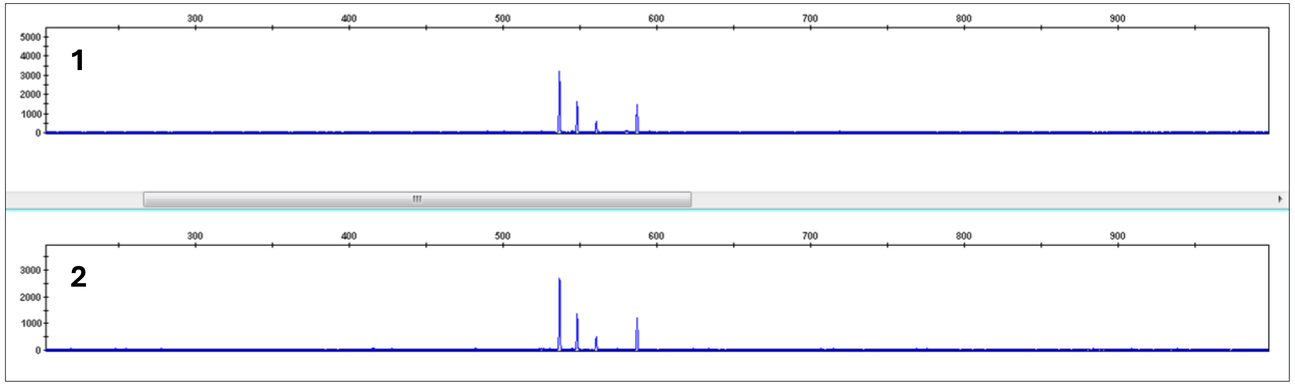

Supplement: Supplementary file 1 [file life-14-00497-s001.zip › life-2814016-supplementary.pdf]
